# Assembly and Rearrangement of Particles Confined at a Surface of a Droplet, and Intruder Motion in Electro-Shaken Particle Films

**DOI:** 10.3390/ma9080679

**Published:** 2016-08-10

**Authors:** Zbigniew Rozynek, Milena Kaczmarek-Klinowska, Agnieszka Magdziarz

**Affiliations:** 1 Faculty of Physics, Adam Mickiewicz University, Umultowska 85, Poznań 61-614, Poland; mkacz@amu.edu.pl; 2 Institute of Physical Chemistry, Polish Academy of Sciences, Kasprzaka 44/52, Warsaw 01-224, Poland; amagdziarz@ichf.edu.pl

**Keywords:** assembly, intruder motion, segregation of particles, electro-shaking, particle film, patchy structures, patchy colloidal capsules

## Abstract

Manipulation of particles at the surface of a droplet can lead to the formation of structures with heterogeneous surfaces, including patchy colloidal capsules or patchy particles. Here, we study the assembly and rearrangement of microparticles confined at the surface of oil droplets. These processes are driven by electric-field-induced hydrodynamic flows and by ‘electro-shaking’ the colloidal particles. We also investigate the motion of an intruder particle in the particle film and present the possibility of segregating the surface particles. The results are expected to be relevant for understanding the mechanism for particle segregation and, eventually, lead to the formation of new patchy structures.

## 1. Introduction

In this article, we study the assembly and rearrangement of colloidal particles confined at the surface of oil droplets, and we also communicate the possibility of utilizing electric fields for the segregation of particles at a surface of a droplet. A method for segregation or separation of the surface particles would likely be useful in fabricating patchy structures, particularly patchy colloidal capsules.

Patchy structures comprise at least two components with different functionalities; therefore, they possess interesting properties. For example, owing to specific interactions between patches, patchy structures can either self-assemble into complex structures or specifically adhere to a surface [1,2,3], guided–align [4,5,6], or self–propel [7,8,9]. Patchy capsules can additionally be used for storage, transportation, and release of cargo species [10]. There are dozens of methods for fabricating patchy particles [11,12,13,14,15], and some of those methods allow fast production of patchy particles in large quantities. In contrast, a large-scale fabrication of patchy colloidal capsules remains challenging; therefore, researchers seek for new methods of efficient production. Thus far, just a few routes to preparing patchy colloidal capsules have been demonstrated, including production by means of microfluidics [5,10,16] and mechanical pipetting [17,18,19]. In both of those, a patchy colloidal capsule is made via the coalescence of two or more droplets, each with different types of particles. It would be more effective if patchy colloidal capsules were made without the need of pairing the droplets and coalescing. Bulk emulsification is the approach that may offer higher material throughput at lower costs, as compared to the abovementioned methodologies. The challenge is to segregate the different particles contained in each droplet of an emulsion. Within this article, we made an attempt to manipulate and eventually segregate different particles located at the surface of one droplet, as a step needed for the formation of patchy colloidal structures.

Manipulation of particles bounded to a surface of a droplet (hereafter called surface particles) can be achieved by employing electric fields. The family of electrokinetic phenomena offers different types of mechanisms for transportation of the surface particles. The motion of particles can be induced either by electric forces that act directly on a particle (e.g., dielectrophoresis [20] or electrophoresis [21]) or by convective flows of liquids that are produced by electric fields [19]. These mechanisms can be used not only for transportation but also for segregation of the surface particles. For a droplet with two kinds of surface particles (a binary mixture), the dielectrophoresis (DEP) can be utilized to separate these particles: particles that may differ either by size or dielectric properties [21,22]. It was also shown that two types of particles located at the surface of a droplet can be separated the synergetic action of DEP and electrohydrodynamic (EHD) flows [23].

Here, we present experimental results that indicate the possibility for segregating particles by electric fields employing EHD flows and electro-shaking of the particle film. In the first part of this work, we further investigate the effect of the electro-shaking of particle film (the mechanism that was partially described in [24]) and provide new experimental results on the amplitude of the surface particle motion as a function of both the electric field strength and its frequency.

In the second part we present the study of motion of individual particle (called the intruder) travelling through the electro-shaken particle film. The intruder particles differ by size (is larger) from the particles comprising the particle film. The particle film has a ring-shape and is initially formed at the surface of the droplet by electric-field-assisted convective assembly [19]. The particle film is composed of densely packed (i.e., nearly hexagonal packing) particles. For allowing the intruder particle to move through the particle film, we electro-shake the particle film causing unjamming of particles forming the film. Thus, the intruder particles can successively (during each electro-shaking cycle) move through the particle film.

In the last part, we present preliminary experiments on particle segregation. For that, we use a dispersion of particles of two different sizes. We observe the segregation of particle, though it takes long time (many electro-shaking cycles) to achieve this.

## 2. Results

### 2.1. Formation of a Particle Film

**Figure 1:**
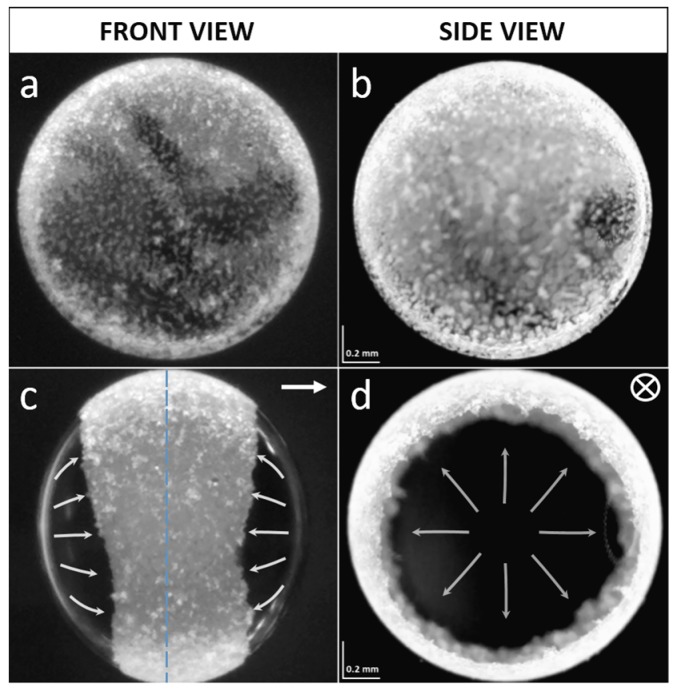
Droplet of silicone oil of diameter ~1.3 mm with PS10 particles. The particles are randomly distributed at the surface of the droplet before the *E*-field is applied (**a**,**b**). After application of the *E*-field of about 250 Vmm^−1^, the particles are guided towards the ‘electric equator’ of the droplet by the EHD flows. The droplet is imaged perpendicular (**a**,**c**) and parallel (**b**,**d**) to the direction of *E*-field. See also corresponding Movie S1.

In this research we study microparticles that are located at the surface of a droplet, where they are bound to the oil–oil interface by capillary force. As a silicone droplet is initially formed in castor oil by pipetting, most of the particles are located inside the droplet, and thus need to be brought to the surface of the droplet. This can be done by any suitable method: for example, by gentle mechanical stirring or by utilizing particle sedimentation due to the density mismatch between the particles and the silicone oil [20,24]. Once all the particles are adsorbed at the surface of the droplet, we assemble them at the electric equator of the droplet to form a monolayered ring-like structure (see Figure 1 and Movie S1). This is done by the action of the EHD flows induced by an *E*-field, as described in [19]. The surface particles form a densely ordered particle film. The particle film remains stable as long as the intensity of the *E*-field does not exceed the critical value; above the critical value, instabilities occur and the ring-like structure breaks apart, forming domains that spin [19], and at very high *E*-fields the particles are removed from the surface of the droplet into the castor oil (see Figure S1). When the *E*-field is turned off, the surface particles may slowly disintegrate from the film starting from those at the boundary of the film (the film either completely ‘liquefies’ or defragments), or it can stay stable; and the behavior of the particle film depends on the types of particles that compose the particle film. We observe that the attractive interactions between the polystyrene (PS) particles themselves are very small, though noticeable, i.e., some of the particles stick to one another, forming short chains (few to several particles long) aligned along the ring, which would be perpendicular to the direction of the *E*-field; and, thus, the particle film defragments rather than completely liquefies. The particle film may remain stable if the attractive interactions between particles are strong, and for example, clay mineral particles adhere to one another rather strongly via tiny water bridges, and such a clay particle film is stable even if the *E*-field is turned off [24].

### 2.2. Electric-Field-Induced ‘Shaking’ of Particles

Shaking of the particle film can be induced by changing polarity of an electric field, and the motion of particles is predominantly in the horizontal direction (along the electric field direction). The mechanism of ‘electro-shaking’ is partially described in [24]. In short, by changing polarity of an *E*-field, a droplet may undergo a shape transition, from being oblate (flatted at the electric poles) to prolate (elongated along the electric poles) and then back to the oblate shape; this occurs due to a redistribution of charges at the surface of the droplet [24]. The particles move slightly upwards or downwards along the ring because the length of the equator changes as the droplet changes its shape. We note that because the PS particles’ dielectric properties are similar to those of the oils, we do not observe particle alignment due to the particle-particle dipolar interactions (within the range of *E*-field strengths used here), as was the case when we used other particles, such as silver spheres [4] or clay minerals [25,26].

If a droplet is placed midway between the electrodes, the EHD flows at the surface of the droplet are roughly symmetrical in respect to the plane of symmetry that bisects the droplet and the particle film in a direction perpendicular to the *E*-field lines, as depicted by the blue dashed line in Figure 1c. Consequently, the induced motions of the electro-shaken particles are roughly symmetric towards/outwards the ring—that is, the particles in the middle of the film move the least, and the particles at either film boundaries move with the highest amplitudes. However, if the droplet is placed at a side of the cell and touches one of the electrodes, the flows of liquid are greatly suppressed on the side where the droplet touches the electrode, hence the EHD flows are not symmetrical anymore. Now, the particles that oscillate with the least amplitude are those at the particle film boundary on the side of the droplet that touches the electrode, whereas the particles on the other side of the film (along the width of the particle film) will have the highest amplitude of motion. In the experiments presented here, we work with a droplet touching one electrode, and there are two reasons for choosing this system: (i) it gives us more control of the droplet that ultimately stays in one location during the experiment; and (ii) we avoid any additional movement of surface particles that may occur because of the motion of the droplet.

The particle behavior during the electro-shaking resembles that of the granular media confined in a two-dimensional cell [27,28,29,30]. Generally, at very low electric fields, the particles within the film remain well ordered and do not relocate. As the intensity of the electric field increases, the particle film undergoes small defragmentation. Further increase of the intensity of the *E*-field causes the film to ‘liquefy’ (though some particles may stick to one another to form chains, which also undergo the motion), allowing the surface particles to relocate. At high *E*-fields, the amplitude of particle oscillation is very large. However, at the compression phase (i.e., at φ→n·180°) the compressed particle film starts to deform (crumple), and at very high *E*-fields, the surface particles may eventually irreversibly detach from the surface of the droplet (see Figure S1).

**Figure 2:**
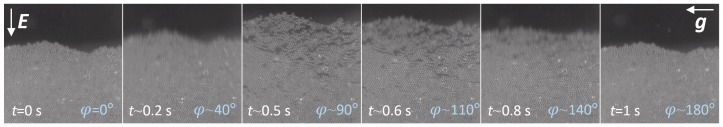
An example of one cycle of the electro-shaking. The AC *E*-field (~650 Vmm^−1^, 0.5 Hz) is in a vertical direction, whereas the direction of gravity is horizontal. See also corresponding Movie S2.

An example of one cycle of the electro-shaking of particles at the surface of the droplet is presented in Figure 2. The AC *E*-field (~650 Vmm^−1^, 0.5 Hz) is vertical, whereas the direction of gravity is horizontal. At φ=0° the particle film is densely packed, whereas at φ=90° it acquires the loosest state, and then at φ=180°, the film is packed again.

**Figure 3:**
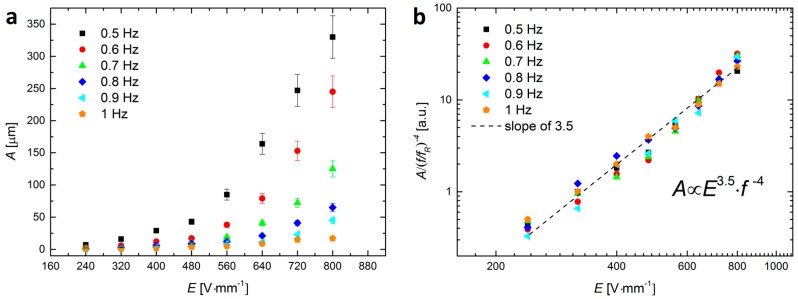
(**a**) The amplitude of particle oscillation versus the intensity of the electric field and its frequency; (**b**) A log–log plot of the data normalized by ${\left(f/f_{R}\right)}^{-4}$, where $f_{R}=1$ Hz is the arbitrarily chosen reference value. The collapsed data roughly follows the dashed line with a slope of 3.5; thus, $A\propto E^{3.5}\cdot f^{-4}$ within the tested ranges of frequency and intensity of *E*-field.

Figure 3a shows the amplitude of particle oscillation A, (calculated as the difference of the positions of the particles at the boundary of the film, at φ=0 and φ=90, respectively) versus the intensity of the electric field, E, and its frequency, f. In order to find out how A scales with E and f, we normalized the data by ${\left(f/f_{R}\right)}^{-4}$, where $f_{R}=1$ Hz is the arbitrarily chosen reference value. The overall data (which collapsed onto each other after normalization) follows the dashed line with a slope of 3.5 (see Figure 3b). Thus, for a droplet sized around 2 mm and with a particle film width of around 0.8 mm and the tested ranges of frequencies (0.5–1 Hz) and strengths of *E*-field (160–800 Vmm^−1^), the amplitude of particle oscillation scales as $A\propto E^{3.5}\cdot f^{-4}$.

Using frequencies higher than 1 Hz is impractical here, since the efficiency of electro-shaking is dropping. This is because at each event of changing polarity of the *E*-field, the droplet needs to charge up and deform. This takes at least 1.2 s for the system of liquids used here [17]. For frequencies lower than 0.5 Hz, and at high intensities of *E*-field, the particle film crumples and particles may detach from the surface of the droplet, as mentioned earlier.

### 2.3. An Intruder Travelling through the Particle Film

A single particle (hereafter called the intruder) of radius *R* was introduced into a sea of roughly monodispersed smaller particles of radius *r* (where *R*/*r* ≈ 14) via electro-shaking. Initially, the intruder was located at the boundary of the particle film. In order to lodge the intruder particle at the film boundary, we electro-coalesced two silicone oil droplets, one with already prepared particle film and the other smaller droplet with an intruder particle situated on the surface of that droplet. The resulting coalesced droplet was subjected to the electric field (square-shaped) of around 600 Vmm^−1^ and with a frequency of 0.5 Hz.

**Figure 4:**
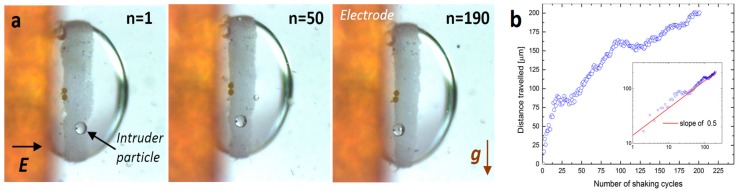
(**a**) Snapshots from the experiment with the PS140 intruder particle travelling through the PS10 film during the electro-shaking. Images taken after 1, 50, and 190 cycles of shaking, from left to right, respectively. Electric field of 600 Vmm^−1^ and 0.5 Hz was in a horizontal direction, whereas the gravitational force was in a vertical direction. See also corresponding Movie S3; (**b**) the distance travelled by the intruder versus the number of shaking cycles. The log–log plot is presented in the inset. The measurement points roughly follow the red line with a slope of 0.5; hence, we may conclude that the data scales as *N*^0.5^.

Figure 4a shows snapshots from the experiment in which the PS140 intruder particle, initially located at the boundary of the film composed of PS10 particles, travels through the film along the direction of shaking, towards the electrode (top part of each image). Figure 4b shows the height of the intruder versus the number of shaking cycles. The data shows that the velocity of the intruder particle moving through the particle film is fastest at the beginning and slowest at the end of the measurement. When the data is plotted in the log–log scale (see the inset of Figure 4b) the measurement points roughly follow the red line with a slope of 0.5; hence, the distance travelled by the intruder scales as *N*^0.5^.

Similar experiments were performed at the DC *E*-field. For the electric field strengths at which the particle film is stable, the intruder particle did not move through the particle film, even if the experiment lasted several minutes. Thus, the motion of the intruder particles was only possible when the particle film was temporarily unjammed due to the electro-shaking.

### 2.4. Size-Segregation of Surface Particles

**Figure 5:**
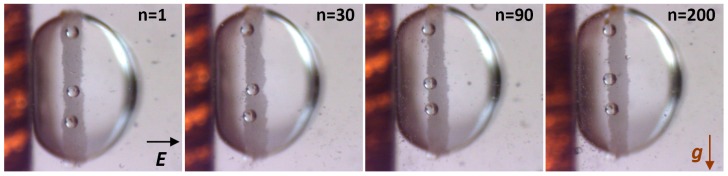
Snapshots from the experiment, in which we attempted to size-segregate the surface particles. Five PS140 particles were initially distributed randomly within the film composed of PS10 particles. Images taken after 1, 30, 90, and 200 cycles of electro-shaking, from left to right, respectively. After about 90 cycles all the PS140 particles were moved to the boundary of the film. Electric field of ~600 Vmm^−1^ and 0.5 Hz was in a horizontal direction, whereas the gravitational force was in a vertical direction. See also corresponding Movie S4.

Figure 5 presents snapshots from the experiment on size-segregating of the surface particles. We prepared a dispersion of PS10 and PS140 particles in silicone oil (with much larger amount of PS10 particles than PS140), and formed a droplet in castor oil. All the steps leading to formation of a ring-shaped particle film were the same as in all above-presented experiments. Before we began to electro-shake (*E* ~ 600 Vmm^−1^, 0.5 Hz) the particle film, five PS140 particles had been distributed randomly within the film. Images shown in Figure 5 were taken after 1, 30, 90, and 200 cycles of electro-shaking, from left to right, respectively. After about 90 cycles all the PS140 particles were moved to the boundary of the film. Further electro-shaking did not change much in the system.

## 3. Discussion

We presented experimental results that indicate the possibility for segregating particles by electric fields employing EHD flows and electro-shaking of the particle film. We studied motion of intruder particles travelling through the electro-shaken particle film composed of particles with smaller size than that of the intruder particles. For allowing the intruder particle to move through the particle film, we were electro-shaking the particle film to unjam particles forming the film and let the intruder particles to pass through the film. We note that the gravitational force was always in a direction perpendicular to the direction of the motion of the intruder particle. Thus, we conclude that the gravity plays no role in the mechanism of the motion of the intruder particle. Possibly the motion of the intruder particle is due to the electric force (e.g., dielectrophoretic or electrophoretic force) acting directly on particles, which is very small in comparison to the force stemming from the EHD flows. Further investigations are needed to provide with a firm understanding of the mechanism of the motion of the intruder particle. Nevertheless, within this communication, we highlight the possibilities of manipulating and eventually segregating different particles located at the surface of a droplet, which can be essential in the process of the formation of patchy colloidal structures. Thus, our results can open new pathways towards the formation of new architectures and structures, such as patchy colloidal capsules or patchy particles. The latter can be produced by solidification of the core liquid after the desired heterogeneous pattern is formed at the surface of the droplet.

## 4. Materials and Methods

The experimental set-up consisted of a sample cell placed on a mechanical x-y-z translational stage, with two digital microscopes for front and side viewing (perpendicular and parallel to the electric-field direction, respectively), a signal generator, a voltage amplifier, an oscilloscope for monitoring signal shape and amplitude, and a PC for collecting images. The sample cell was made of glass with two of the walls coated with a conductive indium tin oxide (ITO) layer, constituting electrodes. The high-voltage bipolar signal was provided to the cell via two crocodile clips attached to the ITO electrodes. The cell was filled with castor oil (83912, Sigma-Aldrich, St. Louis, MO, USA, density of 0.961 gcm^−3^ at 25 °C, electrical conductivity of around 50 pSm^−1^, relative permittivity 4.7, and viscosity of around 700 cSt). A silicone oil droplet containing polystyrene (PS) particles was made in the castor oil using a mechanical pipette (see Figure S2a). In order to minimize the buoyancy force on the droplet with particles, two silicone oils with densities 0.960 and 0.965 gcm^−3^ at 25 °C (200/10 cSt and 200/100 cSt, Dow Corning, Auburn, AL, USA, electrical conductivity approximately 3–5 pSm^−1^, relative permittivity 2.1) were mixed adequately to match the castor oil density. Two batches of the PS particles (PS10 and PS140), with diameters of around 10 µm and 140 µm and specific density of around 1 gcm^−3^, were purchased from Microbeads AS, Skedsmokorset, Norway. The particles were surface modified to change their affinity towards castor oil, which resulted in different contact angles at the castor oil–silicone oil interface, hence influencing the stability of the particles at the surface of the droplet. The acrylate polymer (PFC 502AFA, FluoroPEL™, Cytonix, Beltsville, MD, USA) was used for the surface modification, and methoxy-nonafluorobutane, (7100 Engineered Fluid, 3M™ Novec™, St. Paul, MN, USA) was used as a solvent. The modification steps were as follows: (1) a solution of acrylate polymer and methoxy-nonafluorobutane solvent was prepared in concentration 1:300; (2) the PS particles (20 g) were dispersed in the solution (40 mL); (3) the solvent was removed by using a rotary evaporator, first at 10 min at 50 °C and afterwards at 10 min at 80 °C; (4) the modified PS particles were thoroughly washed with the solvent; and (5) the solvent residues were removed again using the rotary evaporator. The results of such modification are presented in Figure S2b. The high-voltage electric signal was obtained by amplifying a low-voltage signal (SDG1025 Siglent, Nashville, TN, USA) using a high-voltage bipolar amplifier (10HVA24-BP1 HVP, Planegg, Germany). The AC electric signal was always square-shaped and bipolar, and its value was provided as the RMS value (i.e., half of the peak-to-peak value).

## Supplementary Materials

The following are available online at www.mdpi.com/1996-1944/9/8/679/s1. Figure S1: Detachment of surface microparticles form a silicone oil droplet. Initially, the stable particle film was formed using *E*-field of 200 Vmm^−1^, DC. When the polarization of the *E*-field changes and its intensity is increased to more than 800 Vmm^−1^, the particle film undergoes one cycle of shaking. At the compression stage, the compressed particle film starts to crumple and eventually irreversibly detach (some or all particles depending on the *E*-field strength) from the surface of the droplet. The droplet is imaged parallel to the direction of *E*-field through transparent electrodes. The diameter of the droplet is ~1.7 mm. Figure S2: (a) The sample cell was made of glass (15 mm × 15 mm × 30 mm) where two of the walls are coated with electrically conductive ITO layers. The high-voltage bipolar signal was provided to the cell via two crocodile clips attached to the ITO electrodes. The transparent ITO electrodes allow for observation in a direction along the electric field. A droplet containing colloidal particles is made using a mechanical pipette; (b) Modification of the surface chemistry of the polystyrene particles (PS140) resulted in a change of their contact angle at the castor oil–silicone oil interface, Movie S1: Assembly of surface particles by the EHD convective flows, Movie S2: Electro-shaking particle film, Movie S3: Intruder motion through the particle film, Movie S4: Preliminary experiment on particle size-segregation.
