# Supplementary material for: Assembly and Rearrangement of Particles Confined at a Surface of a Droplet, and Intruder Motion in Electro-Shaken Particle Films
Source: Materials (Basel). 2016 Aug 10;9(8):679. doi: 10.3390/ma9080679 (PMC5510736; doi:10.3390/ma9080679)
Supplement: Supplementary file 1 [file materials-09-00679-s001.zip › Materials-137577-supp.pdf]

# Supplementary Materials: Assembly and Rearrangement of Particles Confined at a Surface of a Droplet, and Intruder Motion in Electro-Shaken Particle Films

**Zbigniew Rozynek, Milena Kaczmarek-Klinowska and Agnieszka Magdziarz**

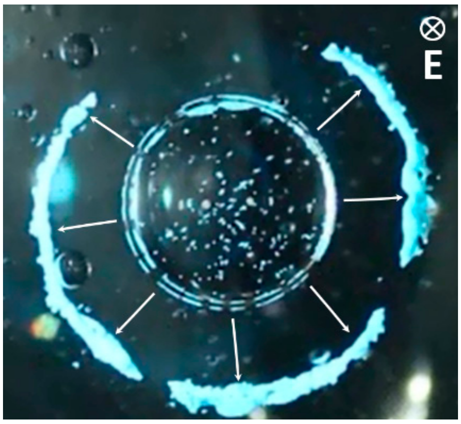


**Figure S1.** Detachment of surface microparticles form a silicone oil droplet. Initially, the stable particle film was formed using *E*-field of 200 Vmm$^{-1}$, DC. When the polarization of the *E*-field changes and its intensity is increased to more than 800 Vmm$^{-1}$, the particle film undergoes one cycle of shaking. At the compression stage, the compressed particle film starts to crumple and eventually irreversibly detach (some or all particles depending on the *E*-field strength) from the surface of the droplet. The droplet is imaged parallel to the direction of *E*-field through transparent electrodes. The diameter of the droplet is ~1.7 mm.

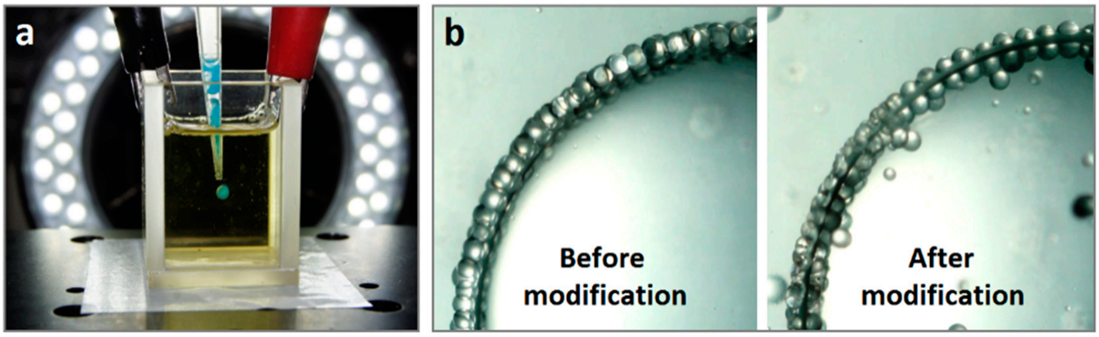


**Figure S2.** (**a**) The sample cell was made of glass (15 mm × 15 mm × 30 mm) where two of the walls are coated with electrically conductive ITO layers. The high-voltage bipolar signal was provided to the cell via two crocodile clips attached to the ITO electrodes. The transparent ITO electrodes allow for observation in a direction along the electric field. A droplet containing colloidal particles is made using a mechanical pipette; (**b**) Modification of the surface chemistry of the polystyrene particles (PS140) resulted in a change of their contact angle at the castor oil–silicone oil interface.
